# Quantum Dots — Characterization, Preparation and Usage in Biological Systems

**DOI:** 10.3390/ijms10020656

**Published:** 2009-02-20

**Authors:** Jana Drbohlavova, Vojtech Adam, Rene Kizek, Jaromir Hubalek

**Affiliations:** 1 Department of Microelectronics, Faculty of Electrical Engineering and Communication, Brno University of Technology / Údolní 53, 602 00 Brno, Czech Republic; E-Mail: hubalek@feec.vutbr.cz (J.H.); 2 Department of Chemistry and Biochemistry, Faculty of Agronomy, Mendel University of Agriculture and Forestry / Zemědělská 1, 613 00 Brno, Czech Republic; E-Mails: ilabo@seznam.cz (V.A.); kizek@sci.muni.cz (R.K.)

**Keywords:** Quantum dots, biosensing, biolabeling, template methods, TiO_2_

## Abstract

The use of fluorescent nanoparticles as probes for bioanalytical applications is a highly promising technique because fluorescence-based techniques are very sensitive. Quantum dots (QDs) seem to show the greatest promise as labels for tagging and imaging in biological systems owing to their impressive photostability, which allow long-term observations of biomolecules. The usage of QDs in practical applications has started only recently, therefore, the research on QDs is extremely important in order to provide safe and effective biosensing materials for medicine. This review reports on the recent methods for the preparation of quantum dots, their physical and chemical properties, surface modification as well as on some interesting examples of their experimental use.

## 1. Introduction

In few past years, researchers in Chemistry and Physics have focused a great part of their interest in fabrication of nanoparticles such as nanowires, quantum dots, nanorods, nanotubes or nanofilms [1]. The reasons for this are the possible applications of nanoparticles in several extremely important fields, e.g. in catalysis, coatings, textiles, data storage, biotechnology, health care, biomedical and pharmaceutical industries. Concerning the medical applications, spherical nanoparticles are the most widely developed and utilized [2]. Nanoparticles can be prepared using metallic, metal oxide–ceramic, polymer, carbon, core-shell, alloy, composite, and biological components.

Nanometer-sized crystals, often referred to as quantum dots (QDs), have been also intensively investigated. Typical QD sizes range between 2–20 nm [3], but according to some literature their diameter should be strictly below 10 nm [4,5]. However, the dimensions of QDs depend mainly on the material used to prepare them. Generally we call a system a QD when the quantum confinement effect occurs, i. e. when the nanoparticle radius *a* is lower than one of this magnitudes: *a*_e_, *a*_h_ and *a*_exc_ (Bohr radius of electron, hole and exciton, respectively). It is known the Bohr radius depends on the material (e.g. 36 nm for InAs, 0.7 nm for CuCl, etc.) and therefore there is not a clear line to say that a nanoparticle is a QD or not, if one only considers its size. QDs can be based on metallic (e.g. Ni, Co, Pt, Au) [6] or (mostly) on semiconductor materials. Moreover, some research into metalloid QDs such as silicon has been done [7]. Because of their reduced size, QDs behave differently from bulk solids due to the quantum-confinement effects that are responsible for their remarkably attractive properties. In general, quantization effects in semiconductor structures can be divided into three groups, depending on whether the charge carriers are confined in one, two or three dimensions [8]. Confinement in one direction creates two-dimensional (2D) structures that have been termed quantum wells or quantum films. Carrier confinement in two directions produces one-dimensional (1D) quantum wires and confinement in three dimensions produces already mentioned quantum dots or quantum boxes, where the electrons are mostly localized; these structures are zero-dimensional (0D). Typically, QDs are represented by atomic clusters or nanocrystallites. QDs usually consist of few hundreds to a few millions of atoms, but only a small number of electrons (≤100) are free [9]. In addition, depending on electron confinement, it is possible to distinguish between planar, vertical and self-assembled QDs, whereas the very first experiments were made with the planar structures. In planar and vertical QDs, electrostatic confinement leads typically to dimensions around 100 nm and structural confinement is of the order of 10 nm, while in self-assembled QDs the structures are rather pyramidal or lens-shaped with sizes of approximately 10 nm. These pyramidal QDs are very promising for laser applications [10,11].

## 2. Quantum dots usage

### 2.1. The main criteria for using QDs in Medicine

Unfortunately, most QDs are toxic, which implies a potential danger, especially for future medical applications. The most widely used and studied QDs consist of a core of cadmium selenide or telluride, because their quantum confinement region spans the entire optical spectrum [12]. Walling and colleagues reported in their review on *in vivo* QDs application that cadmium ions were determined as the primary cause of cytotoxicity, because they are able to bind to thiol groups on critical molecules in the mitochondria and cause enough stress and damage to cause significant cell death [13]. However the release of Cd^2+^ could be reduced or eliminated by adding additional surface coatings. Scientists in Ireland have been using gelatin during the production of CdTe QDs, thereby reducing the toxicity of the particles [14]. In other case, Qian *et al.* used as stabilizer the tripeptide glutathione, which exists in most organisms and thus can be applied to detoxify Cd^2+^ ions in Medicine due to its chelating capability [15]. Iyer *et al.* reported that peptides could be used as an excellent ligand for coating the surface of QDs for several reasons: 1) peptides mimic the biological environment and are stable at physiological pH; 2) reactive groups (amines, carboxyl, thiol, and peptide tags) can be dialed into the hydrophilic domain of the peptide sequence and enable enzymatic or standard conjugation chemistry to obtain biomolecules of interest; 3) molecular evolution strategies to randomize peptides can be adapted to select high-affinity binders to the QD surface; and 4) there is the possibility of creating multifunctional QDs by mixing peptide sequences in certain molar ratios in a single step for *in vitro* and *in vivo* studies (e.g., a QD with biotin and PEG, conjugating receptor ligand and PEG, etc.) [16].

In other words, surface functionalization plays the key role in nanoparticle toxicity. It was found the improved biocompatibility that certain surface coatings provide could arise from decreased cellular uptake of these nanoparticles for cellular degradation [17]. According a review of Hardman, QD absorption, distribution, metabolism, excretion and toxicity depend on multiple factors derived from both inherent physico-chemical properties and environmental conditions; QD size, charge, concentration, outer coating bioactivity (capping material and functional groups), and oxidative, photolytic, and mechanical stability have each been implicated as determining factors in QD toxicity [18]. If this toxicity problem could be addressed, QDs might one day be safely used as fluorescent probes for biological imaging, to monitor targeted drug delivery and for controlled modification of structural and functional properties of intracellular components. An interesting study in this field was performed by Choi and colleagues, who examined the epigenomic and genotoxic response to cadmium telluride QDs in human breast carcinoma cells [19]. They suggest three levels of nanoparticle-induced cellular changes: non-genomic, genomic and epigenetic. Epigenetic changes may have long-term effects on gene expression programming long after the initial signal has been removed, and if these changes remain undetected, it could lead to long-term untoward effects in biological systems.

According to many studies, short-term (acute) and long-term toxicity of QDs is an important issue for QD applications in bio systems; not only at the cellular level, but also in animal models [20–22]. Cell death caused by cadmium ion release is not the only type of toxicity exhibited by nanoparticles. Quantum dots can also damage DNA and disrupt normal cell activity caused by factors such as the surface coatings themselves [13]. Geys et al. determined the acute *in vivo* toxicity of QDs with carboxyl and amine surface coatings during investigation of the inflammatory properties, tissue distribution, and prothrombotic effects after intravenous injection into mice [23]. Yong demonstrated that doping of QDs by manganese and their subsequent surface functionalization with lysine makes them stably disperse in aqueous media and moreover these QDs, emitting in the near-infrared (NIR), reveals no long-term toxic effects when injected to mice body [24]. Therefore he supposed these multimodal Mn-doped QDs have potential as probes for early pancreatic cancer imaging and detection. Chen and colleagues observed the viability, lifespan, behavior and health of the mice after intravenous injection of thiol-capped CdHgTe QDs for three months [25]. They found that injection of 2 μg/g CdTeHg QDs did not exhibit significant toxicity, and no abnormal behavior of the mice was observed in any of the *in vivo* imaging experiments. Nevertheless, a key question is still open: whether QDs can be used directly in clinical phase studies? Consequently integrated cellular toxicity studies and the complete *in vivo* toxicology of QDs still need to be evaluated before their use in human applications.

The other important criteria for QDs using in medicine are prevention of nanoparticle aggregation in a biological environment and effective suppression of non-specific adsorption of biomolecules at the nanoparticle surface. The insufficient colloidal stability can be improved by functionalization of QDs surface with hydrophilic polymers such as PEG [26] or with adsorbed peptides [27]. Conveniently, the long-term stability of QDs can be improved by storing them in lyophilized composition, in the absence of light and at reduced temperature (e.g. at 4 °C) [28].

In contrast to organic fluorophores, QDs have some unique photophysical properties. They have a continuous absorption spectrum for wavelengths shorter than the wavelength of fluorescence emission. QDs can be synthesized to be effectively monodispersed which is a cause that their emission spectra are quite narrow and symmetric, and do not show any red-tail. In this way many different colors can be excited with just one wavelength of excitation and can be spectrally well resolved [29]. For example, Liu and colleagues employed traditionally used fluorescence resonance energy transfer technique (FRET) to study the molecular interaction between an antibody and immunoglobulin G (IgG) on live cell membranes using QDs of two different colors, green and red, which were used as FRET donors and acceptors, respectively, at the same time without organic dyes being involved [30]. The dots are claimed to be 20 times as bright and 100 times as stable against photobleaching, when compared to conventional fluorophores [31].

However there are two disadvantages in comparison to classic fluorescent dyes. The first negative point of QDs is the lack of strong polarization of the emitted photons, mainly in the case of elongated QDs, also called nanorods [32]. The second shortcoming is their insolubility in water. This insolubility is generally caused by the use of organic solvents (e.g. trioctylphosphine oxide, TOPO) for their preparation and hence by the presence of hydrophobic molecules. This problem can be solved by embedding of QDs into a shell (e.g. silica) or stable surfactant layer.

As it is known, the photoactivation of the semiconductor nanoparticles is highly affected by several parameters. Dembski and colleagues have systematically investigated the role of those parameters on the photon-induced photoluminescence enhancement of CdSe/ZnS QDs embedded in silica colloids (multicore particles) and polymer-stabilized CdSe/ZnS QDs in various environments [33]. The studied parameters included the local environment of the QDs (polymer shell or silica matrix), the thickness of the outer silica shell of the multicore particles, the dispersion medium, as well as the wavelength and intensity of the incident radiation. Zhang *et al*. also found the photoluminescence lifetime of intracellular thiol-capped CdTe QDs varied greatly depending on the intracellular environment: in the acidic lysosomes (with a pH of around 4.5–5.0) was remarkably shorter than those in other parts of the cell [34]. On the other hand, the photoluminescence lifetimes of QDs in living cells were generally shorter than those in aqueous solutions with similar pH. Therefore the acidic environment and the interactions of QDs with biomolecules are two of the possible reasons for the shortening of photoluminescence lifetimes of intracellular QDs. It is also known that the II–IV semiconductor QDs photoluminescence depends on temperature: as the temperature increases, the photoluminescence peak of QDs redshifts. Despite this fact, Zheng’s group observed that in the temperature range of 120–300 K, the photoluminescence peak of CdSe QDs initially blue-shifts and then red-shifts with increasing temperature arising from the thermally activated detrapping of carriers [35].

### 2.2. Some important applications of QDs

**Figure 1:**
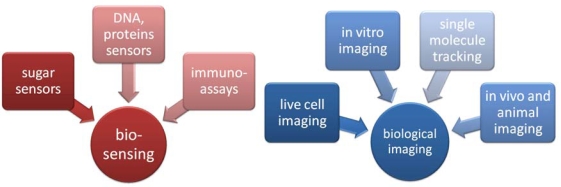
Examples of QDs’ bioanalytical and biomedical applications.

Concerning the biological applications of QDs, two main groups may be cited: biosensors and labels in biological imaging. A few examples of each group can be seen on the schema below (Figure 1).

QDs are excellent candidates for biosensing owing to their unique physical and optical properties and possibility of attaching various biomolecules to their surface [36]. Some new assays of QDs’ usage, which can improve the current methods of DNA and protein detection, were performed [37, 38]. For example, a detection method of adenosine-triphosphate (ATP) using a QD tagged aptamer (nucleic acids that bind to certain molecular targets such as thrombin, adenosine, or cocaine) has been described [39].

Most biomolecules have been linked to water-soluble QDs and it was found the binding has no effect either on the QDs’ optical signature or on the functionality of the biomolecules. Biomolecules can be bound to the surface of QDs either directly (covalently or non-covalently) or attached via a stabilizing layer which act as a crosslinker between the ligand and reactive surface of the nanoparticle. Non-covalent direct binding can be achieved by applying of electrostatic-based coupling strategy. Compared to the conventional covalent techniques, the electrostatic non-covalent self-assembly approach is simpler, more reproducible, and more easily achieved [40]. This approach has been used for example to bind cysteamine-stabilized CdTe QDs with single stranded DNA through electrostatic attraction between positive amino function groups on the surface of CdTe quantum dots and negatively charged DNA [41].

The covalent bioconjugation approach is based on the replacement of thiol acids present on the QDs’ surface with thiolated biomolecules. This type of binding was chosen, for example, to covalently link streptavidin maleimide [42] or to conjugated transferrin and mouse anti-human CD71 monoclonal antibody to CdSe/ZnS QDs [43]. Use of water-soluble 1-ethyl-3(3-dimethylaminopropyl) carbodiimide hydrochloride (EDC) and *N*-hydroxysulfosuccinimide (NHS) to form the QD-protein covalent conjugates is one of the most frequent coupling methods. The research group of Wang determined that 3-mercaptopropyl acid-stabilized water-soluble CdTe nanoparticles synthesized in aqueous solution were able to conjugate with peptides or proteins mediated by NHS [44]. These stable QDs peptides/protein conjugates can provide great potential power in cell labeling application.

**Figure 2:**
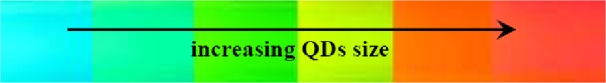
An example of QDs sorted by size emitting light of different colors excited simultaneously by a single excitation wavelength.

In these applications, core-shell structured QDs are more favorable, where CdSe is referred to a core and ZnS to a shell [45–47]. The dimension of the core determines the bandgap and hence the color of emission. It is known, that an increase in particle sizes produces a redshift in the emission spectrum [48]. In principle, the emission of QDs can be coarse-tuned by the choice of the material and later fine-tuned by playing with the size of the core (see Figure 2). The emission color can be also tuned from the UV/blue spectral region (ZnSe) to the visible one (CdSe) by changing the composition, that is, the Cd:Zn ratio, without changing the nanocrystal size [49]. Many core-shell QDs have been prepared by capping an emissive semiconductor core (CdSe, CdTe, etc.) with a thin shell of a higher band gap material (ZnS, CdS, ZnSe, etc.) [50–52]. For example, the core-shell CdSe/ZnS are about 20–50× brighter than CdSe cores and their quantum yield can reach 30–50%.

There are several possibilities for using QDs in biolabeling and cellular imaging both *in vitro* and *in vivo*. Scientists have developed hybrid functionalized QDs-liposome nanoparticles and found they were efficiently uptaken by living cells in the absence of cell death and can therefore be used as fluorescent probes for *ex vivo* cell-labeling studies with most types of water-soluble QD without further modifications [53]. Moreover, these QDs exhibited enhanced penetration and retention into the tumor interstitium both *in vitro* (tumor spheroids) and *in vivo* (subcutaneous solid tumors). Pan and colleagues evaluated *in vitro* QDs loaded in poly(lactide)-vitamin E TPGS (d-α-tocopheryl polyethylene glycol 1000 succinate) nanoparticles for cellular and molecular imaging [54]. Kerman *et al.* studied an application of QD-labeled biomolecules in a sandwich-type immunoassay for detection of total prostate-specific antigen (TPSA), which is an important cancer marker, on a screen-printed carbon substrate in connection with fluorescence imaging [55]. Similar QD-label-based electrochemical immunoassay study of PSA was performed by Wang and colleagues [56].

**Figure 3:**
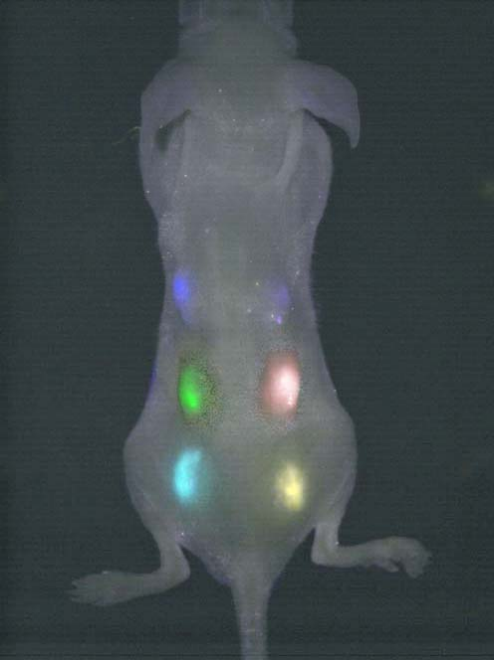
Embryonic stem cells labeled with six different QDs were subcutaneously injected on the back of the athymic nude mice right after labeling (image taken with a single excitation light source right after injection). Reprinted with permission from [62].

QDs can be applied in molecule tracking in immunochemistry, where they replace the fluorescent beads used for the study of the dynamics of neurotransmitter receptors. Due to their much smaller size (about 10–20 nm) compared to latex beads (approx. 500 nm), the lateral movement of individual receptor can be studied in great detail [57]. Another example of an application of QDs is in genetic disease screening and diagnostics, where in combination with stage-scanning confocal microscopy provide the imaging of QDs free of chromatic aberrations and with resolution better than 10 nm [58, 59]. Infrared QDs were also found to be useful probes for non-invasive detection *in vivo*, mainly inside small animals, where they can substitute conventional organic fluorophores emitting in the IR, which suffer from poor stability and quantum yield [25, 60, 61]. Lin *et al.* evaluated *in vivo* multiplex imaging of mouse embryonic stem (ES) cells labeled with peptide-based Qtracker delivered QDs [62] (see Figure 3.). They show that labeling mouse ES cells with QDs does not adversely affect ES cell viability, proliferation, and differentiation.

Further utility of QDs have been found in labeling of nucleus in live cells, however this issue has not been not fully studied [63]. For example, Chen and Gerion observed, that the viral peptides called nuclear localization signals conjugated with CdSe/ZnS QDs has no toxicity effect in HeLa cells transfected with the peptide-coated QDs [64].

Lieleg and collegues have developed a method of specific labeling of membrane integrins in living osteoblast cells using functionalized QDs [65]. They used cyclic Arg-Gly-Asp (RGD) tripeptide sequence and a biotin–streptavidin linkage to specifically couple individual QDs to integrins of living cells. However, they noticed a drastic decrease in the total number of blinking QDs during observation. Fortunately, according to the simulations, the blinking properties of QDs do not harm the quantitative evaluation of the obtained integrin trajectories.

It has been proven that QDs can serve as luminescent cell markers that identify molecular structures. Generally, effective multicolor cell labeling using QDs can be achieved via receptor-mediated uptake or via nonspecific endocytosis which invokes native cellular mechanisms to transfer nanoparticles through the cell membrane. Hence endocytosis is considered to be the least disruptive delivery method compared to conventional methods such as microcapillary injection or electroporation, which are based on inserting the nanoparticle cargoes via microscopic mechanical defects in the cell membrane [66].

## 3. Quantum dot preparation and characterization

### 3.1. QD preparation

Two general approaches for the preparation of QDs have been reported over the last decade: (1) formation of nanosized semiconductor particles through colloidal chemistry [67,68] and (2) epitaxial growth and/or nanoscale patterning [69, 70], i.e.. employing lithography-based technology. The former QD synthesis relies on rapid injection of semiconductor precursors into hot and vigorously stirred specific organic solvents containing molecules that can coordinate with the surface of the precipitated QD particles. This synthetic route seems to be facile and can be performed in “one-pot” as referred in many papers [15,71–73]. QDs designed for usage in biological systems are mostly applied in solution (colloidal form); nevertheless a demand for QDs deposited on various solid surfaces for biomedical applications was also emphasized in some papers [69,74]. For example, an alternative and promising strategy for the use of QDs in biotechnological applications is to utilize biofunctional carrier spheres that are labeled with QDs [75].

As mentioned before, QDs need to be water-soluble. Therefore numerous effective methods have been developed for creating hydrophilic QDs, which can be divided into two main categories [66, 76]. The first route is commonly designated as “cap exchange”. The hydrophobic layer of organic solvent can be replaced with bifunctional molecules containing a soft acidic group (usually a thiol, e.g. sodium thiolycolate) and hydrophilic groups (for example carboxylic or aminic groups) which point outwards from the QDs surfaces towards bulk water molecules [77–79]. In fact, substitution of monothiols by polythiols or phosphines usually improves stability. The second route is native surface modification, for example, adding of a silica shell to the nanoparticles by using a silica precursor (usually alkoxysilanes such as tetraethylorthosilicate, TEOS) during the polycondensation [80]. Amorphous silica shells can be further functionalized with other molecules or polymers. Another example can be introducing of amphiphilic molecule, such as a phospholipid. This procedure is preferred for commercially-produced biocompatible QDs. The method of QD encapsulation into solid lipid nanoparticles, which are composed of high biocompatible lipids of physical and chemical long-term stability, was also successfully tested [81]. These lipid nanoparticles are more convenient than small molecules (e.g. mercaptopropionic acid) traditionally used for QDs surface modification, which are rather unstable since they can be easily degraded by hydrolysis or oxidation of the capping ligand. To make water-soluble QDs, Zhang *et al*. employed tricopolymer coating with the oil in a water ultrasonic emulsification method, and demonstrated the binding assays based on QD–antibody bioconjugates [82]. Ultrasonication will accelerate evaporation of the organic solution (in this case dichloromethane), which will shorten the preparation time.

**Figure 4:**
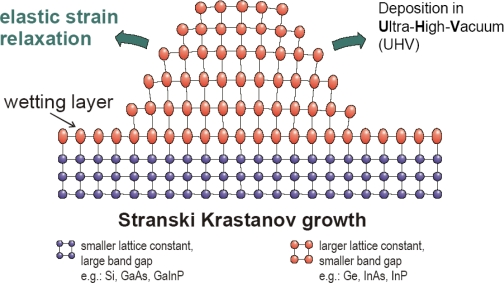
Schema of self-assembled quantum dots growth. Reprinted with permission from [86].

The latter approach of QD preparation, lithography based technology, is widely used to provide QDs predominantly by the combination of high-resolution electron beam lithography and subsequent etching [10,83]. However, it was found the spatial resolution required for reaching the size regime, where significant quantization effects can be expected tends to be larger than the desirable level. In addition, lithographic methods and subsequent processing often produce contamination, defect formation, size non-uniformity, poor interface quality, and even damage to the bulk of the crystal itself [84]. Finally, it was found that traditional top–down patterning methods like photolithography and e-beam lithography are time-consuming and expensive processes, therefore there is a demand for new more sophisticated techniques for QDs fabrication [85]. One of these techniques can be an epitaxial growth of nanostructures, which is generally described by 5 various modes. The fabrication of epitaxial QDs usually follows the Stranski-Krastanov mode of growth on wetting layer with formation of coherent islands (see Figure 4). Here, no further etching process is required.

Epitaxial method of QDs preparation is widely used in optoelectronics (lasers, infrared photodetectors) and nanotechnologies. However, in the near future, it could be very interesting and promising to use epitaxially grown QDs in *in situ* biosensing, mainly due to the simplicity of detection, for example, as a sensor array for mass screening. Thanks to this sensor arrangement, where each sensor can be created from QDs emitting light at different wavelengths, it could be possible to easily detect many different biomolecules at the same time. Epitaxial methods can be classified according to phase origin into vapor phase epitaxy (VPE) or liquid phase epitaxy (LPE). Although LPE is still important, this technique was used mostly in past century to prepare micrometric structures. VPE can be further categorized according to reaction type into chemical (CVD) or physical vapor deposition (PVD). Chemical deposition is interesting in view of its price, however the most important and most widely used technique in industry is VPE from metalorganic medium (MOVPE). Molecular beam epitaxy (MBE) is other peculiar PVD technique and can be also classified into solid source MBE or gas source MBE (hydride or metalorganic). Perhaps less well-known approach for the growth of nanostructures using MBE system is “droplet epitaxy” [87]. Here liquid metal droplets are first formed as an intermediate growth step before being converted into semiconductor nanostructures.

Concerning the QDs direct patterning, self-assembled monolayers QDs can be coated via Langmuir-Blodgett technique onto hydrophobic–hydrophilic pattern generated with employing of photolitographically prepared template [69].

For the past few years, template-based nanoengineering methods, in which many different materials such as porous alumina, polymer gel, surfactant, activated carbon and carbon fiber have been used as templates to synthesize different kinds of nanostructured porous materials, have been extensively studied. For example Ghanem *et al.* prepared metal nanodots using the electrochemical deposition through cavities formed within macroporous poly(pyrrole) secondary templates which were themselves prepared by electrodeposition around templates formed of self-assembled poly(styrene) spheres assembled on evaporated Au substrates [6]. Recently, a “microscopic mass-point addition” method was developed to fabricate high density TiO_2_ nanodots with controllable size on a Si substrate, where an alumina filtration membrane with homogenous micropores acts as the “microscopic mass-point” nozzle [38]. The titania nanostructures mimic the ordered nanoporous anodic alumina films via the through-mask anodization [88]. During anodic oxidation of titanium foil, different electrolytes can be used, like HF, KF, NH_4_F with ethylene glycol [89].

The phase development of the isolated TiO_2_ nanodots is very much different from that of TiO_2_ thin films and powders. After high temperature annealing, the nanodots are polycrystalline and consist of a mixed phase of anatase and rutile instead of single rutile phase. Scientists expect that TiO_2_ nanodots with a single phase of anatase can be prepared from an epitaxial Al/TiN bilayered film on a sapphire substrate by electrochemical anodization of the TiN layer using a nanoporous anodic aluminum oxide film as the template [90]. The anatase/rutile phase composition influence on TiO_2_ QDs properties is very complex, because several parameters affect the final quantum confinement (the multiple influence of QDs size and coordination of Ti atoms on Ti–O bond length and thus on band gap energy) [91,92].

The use of non-toxic titanium dioxide can completely suppress the problem of QDs toxicity in biomedicine for *in vitro* applications. The enhanced photocatalytic effect of TiO_2_ nanoparticles in skin and other types of cancers was recently demonstrated [2]. Oligonucleotide DNA covalently attached to the TiO_2_ nanoparticles was shown to have a unique property of a light-inducible nucleic acid endonuclease, which could become a new tool for gene therapy. The preparation of TiO_2_ nanodots with small dot size and high dot density on substrates becomes increasingly significant since small dot size can guarantee quantum effects and high activity, while high dot density can provide a basis for miniaturizing devices and increasing performance capacity [93]. It has been proven that the modification of TiO_2_ on the electrode could enhance the enzyme catalytic performance for promising biosensor applications. However, the electrochemical behavior of TiO_2_ depends not only on the crystal structure and surface properties, but also on the textural properties, which include specific surface area, pore volume, pore dimension and distribution. Great effort has been dedicated to the synthesis of TiO_2_ with a controllable morphology and pore structure [94]. Scientists also investigated a rutile TiO_2_ QDs electronic structure via the first-principles band structure method [92].

### 3.2. QD characterization

Optical characterization of QDs is usually provided by UV-VIS and photoluminescence spectroscopy, which offer fast, non destructive and contactless option. Hazdra and colleagues employed a rarely used experimental method, photomodulated reflectance spectroscopy, for checking deposited QD structures. This technique provides equivalent energy resolution to that of photoluminescence at low temperatures and probes a wider range of critical points, highlighting the ground state and also many high order interband optical transitions from which band structure, especially of the wetting layer, can be deduced [95].

As already mentioned, the optical properties (fluorescence emission) of QDs can be fine-tuned by the QDs’ size, which is a key parameter that determines the spectral position and purity of photoluminescence. QDs’ size is generally calculated using conventional techniques like scanning electron microscopy (SEM), transmission electron microscopy (TEM), and dynamic light scattering (DLS) studies. Gu and colleagues determined the size and composition of optically active CdZnSe/ZnBeSe QDs using photoluminescence, photoluminescence excitation, and Raman scattering spectroscopies combined with a model of photoluminescence and LO phonon energies [96]. Besides these techniques, field flow fractionation was also successfully employed an excellent complement to characterization of water soluble QDs by the conventional tools [97].

**Figure 5:**
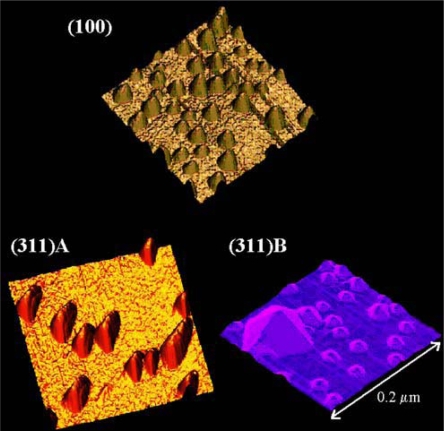
STM image of InAs/GaAs QDs grown by MBE on GaAs. Reprinted with permission from [84].

In order to monitor the size of epitaxially prepared QDs, several characterization methods are usually employed, e.g. TEM, atomic force microscopy (AFM) or more preferably scanning tunneling microscopy (STM) as can be seen from Figure 5. Scientists also evaluated InAs QDs’ size from magneto-tunnelling experiments and they found the results in very good agreement with the sizes measured by AFM and TEM [98]. In order to study quantum size effects on the electronic structure of InAs and InGaAs QDs in correlation with their morphologies, scanning tunneling spectroscopy was used in the work of Yamauchi [99].

According to Lees *et al*., the QDs’ surface chemistry plays also very important role, because it contributes to the final hydrodynamic diameter of QDs and thus it is necessary to find a method providing qualitative information about this parameter [100]. To date a number of different techniques have been employed to characterize QD surface chemistry, notably X-ray photoelectron spectroscopy, nuclear magnetic resonance spectroscopy, and Rutherford backscattering. Lees *et al.* characterized the surface chemistry and size distribution of ODs using analytical ultracentrifugation method, which was found to be highly sensitive to nanocrystal size, resolving nanocrystal sizes that differ by a single lattice plane.

## 4. Conclusions

QDs have been found useful due to their sensing capability for various particular applications, in fields such as optoelectronics and medicine. The main reasons for their use for medical purposes are the following: excitation wavelength far from the emission, inherent photostability and long fluorescent lifetime. It is evident that an appropriate material for biosensing devices will be very different from the one suitable for optical devices. In particular the question of QDs’ toxicity in the former applications is very important, mainly for essays performed *in vivo*. Therefore scientists are making great efforts to solve this problem, generally by coating QDs surface with protecting and stabilizing shell, in order to use them safely for subsequent bioconjugation with proteins, peptides or other chemical moieties. It was also found that round shape of QDs is preferred when using them in solution for biosensing, so called colloidal QDs, as it was referred in majority of papers, while the deposited QDs with shape of islands are generally required for optoelectronic media.

Concerning the medical applications, there are still several issues, like desirable orientation of attached biomolecules, which remain to be resolved, so it can appear that commercial QDs are more appropriate for some approaches. Hence, it is necessary to continue the QDs research to develop their surface functionalization and conjugation with biomolecules more precisely. From biomolecules detection point of view, it is also interesting to study the behavior of QDs immobilized on variety of supports.
